# Intrahepatic Cholangiocarcinoma—Where Are We Now and Where Are We Going to?

**DOI:** 10.3390/medicina59040729

**Published:** 2023-04-07

**Authors:** Michał P. Wasilewicz, Rafał Becht

**Affiliations:** 1 Liver Unit, Department of Gastroenterology, Pomeranian Medical University in Szczecin, 71-252 Szczecin, Poland; 2 Clinical Oncology, Chemotherapy & Immunotherapy of Tumors Unit, Pomeranian Medical University in Szczecin, 71-252 Szczecin, Poland

Cholangiocarcinomas (CCAs) are a heterogeneous group of malignancies originating from the biliary tract epithelium. Based on the location of the lesion (according to the anatomical division of the bile ducts), we distinguish:

1. Intrahepatic cholangiocarcinoma (iCCA)
2. Extrahepatic cholangiocarcinomas:

(a) perihilar cholangiocarcinoma (pCCA; formerly called Klatskin tumor)—located in the vicinity of the hepatic hilum;
(b) distal cholangiocarcinoma (dCCA)—located in the distal section of the common bile duct.

Due to its specificity of formation and natural course, gallbladder tumors constitute a separate group [1,2,3,4,5,6].

Intrahepatic cholangiocarcinomas account for 15–20% of all bile duct malignancies and have a very poor prognosis [1]. The mortality rate of patients with cholangiocarcinoma, regardless of geographical region, is high, and has basically not changed significantly for many years. In total, the 5-year overall survival (OS) rate of patients with this disease does not exceed 10% and has remained at a similar level for many years. This is mainly because most patients are diagnosed with the disease at the time of significant clinical advancement. This is related to the oligosymptomatic course of the disease. Early cholangiocarcinoma is usually detected incidentally in different imaging procedures performed for reasons other than a suspected focal lesion in the liver. A typical example is searching for the cause of abnormal laboratory liver test results, especially elevated cholestatic parameters. By the time clinical jaundice or weight loss occurs, iCCA is usually much more advanced. Unfortunately, in the last few decades, we have observed a significant increase in the incidence of this type of cancer worldwide (nearly 15% more new cases per year) [6].

ICCA has a close relationship with the ongoing process of chronic inflammation in the bile ducts and the surrounding liver parenchyma, especially with the accompanying chronic cholestasis. This situation is associated with chronic damage to the cholangiocytes, and during the process of their regeneration, an error may occur at some stage of the process, starting the formation of a cancer cell line. Therefore, the common risk factors for biliary tract cancer include diseases, such as: cystic biliary tract disease, primary sclerosing cholangitis, intrahepatic cholelithiasis, liver cirrhosis (especially in the course of chronic viral hepatitis, alcohol abuse or metabolic-associated fatty liver disease (MAFLD)), parasitic infections of the bile ducts and exposure to certain chemical agents, including nitrosamines or significant iron load (e.g., in hereditary hemochromatosis (HH)) [2,4]. It has also been proven that iCCA may be associated with some genetic diseases, such as Lynch syndrome (hereditary non-polyposis colorectal cancer (HNPCC)), cystic fibrosis, mutations of BRCA-1-related proteins or the abovementioned HH, although spontaneous cases with no clearly established risk factors predominate [1,5].

The gold standard in the diagnosis of iCCA remains contrast-enhanced radiological imaging methods, such as multiphasic computed tomography (CT) or magnetic resonance imaging (MRI) of the abdomen, which simultaneously allow for the assessment of the extent of the disease process and the potential resectability of the iCCA lesion. A complementary procedure to radiological imaging, allowing for the final diagnosis of iCCA, is a core-needle biopsy of the liver tumor. This offers the opportunity to state definite diagnosis with a thorough histopathological and pathogenetic assessment in terms of, for example, the availability of potential new treatment regimens using targeted immunochemotherapy. Ca 19-9 and CEA markers may be of some importance in the diagnosis, but the specificity and sensitivity of their presence in iCCA at a level not exceeding 65% does not allow us to use them as one of the main diagnostic tools [5,6].

Surgical treatment (R0 resection with a margin of normal tissue) has been the only possible radical treatment of iCCA for many years [2]. However, only about 25–30% of patients can be offered such therapy due to the stage of the disease at the time of diagnosis [5,6]. In addition, the natural course of the disease shows that patients qualified for potential radical surgery have a 5-year OS rate not exceeding 35–40% [1,7]. That is why, after the publication of two significant clinical trials (*BILCAP and PRODIGE 12-ACCORD 18*), which clearly demonstrated improvements in CCA treatment results, the therapeutic scheme for patients was supplemented with postoperative systemic chemotherapy with capecitabine and, nowadays, it is included in the standards of management for patients with iCCA [2,4]. Further, patients with unresectable iCCA at the time of diagnosis, thanks to the use of capecitabine, extended their average survival period to nearly 12 months [5]. For patients with the most advanced forms of the disease, palliative chemotherapy using gemcitabine and cisplatin is recognized as the standard of first-line treatment [8]. However, in the light of the latest research, the use of targeted immunochemotherapy in combination with classic gemcitabine/cisplatin chemotherapy (or even immunotherapy alone) may soon change the iCCA treatment guidelines. A very good example of an increasingly favorably used drug in such a therapeutic combination is durvalumab, administered according to the scheme known from the TOPAZ-1 study [3,8]. Locoregional therapies, such as trans-arterial chemo- and radioembolization (TACE/TARE) or radiofrequency ablation (RFA), remain a certain therapeutic option in palliative management in patients with unresectable iCCA. Liver transplantation may sometimes be appropriate, in a very restrictedly selected group of patients with a tumor not exceeding 3 cm diameter. However, the very low rate of early diagnosed patients explains the low applicability of this method in clinical practice [1,5,7].

In recent years, along with the development of molecular research in modern clinical oncology, significant progress has been made in iCCA research. Many of the common genetic mutations present in iCCA tissue have been identified. It is currently believed that up to 70% of patients with iCCA may have molecular disorders in their cancer cells, which may be a potential target for modern targeted immunochemotherapy. The most frequently identified genetic alterations in iCCA cells include aberrations in genes encoding proteins: TP53, KRAS, SMAD4, MET, EGFR, FGFR2, IDH1/2, RB1, ERBB2 or BAP1 [8,9,10]. According to the studies carried out so far, it is believed that lesions positive for EGFR, ERBB2 or KRAS mutations in their cells are associated with a more aggressive course of the disease, which obviously worsens the prognosis of patients [6]. One of the most recent discoveries, which may be of great importance in iCCA-targeted therapies, is genetic FGFR2-G3BP2 fusion [11,12,13]. This is why, now, due to the identification of numerous genetic abnormalities in iCCA cells, there are many potential opportunities for the use of new drug molecules that remain in clinical trials (pemigatinib, futibatinib or ivosidenib) [1,7,12]. It is likely that complementary personalized systemic targeted immunochemotherapy will be a new treatment alternative for patients with iCCA in the coming years [3,4].
